# The Slowly Aggregating Salmon Calcitonin: A Useful Tool for the Study of the Amyloid Oligomers Structure and Activity

**DOI:** 10.3390/ijms12129277

**Published:** 2011-12-13

**Authors:** Marco Diociaiuti, Maria Cristina Gaudiano, Fiorella Malchiodi-Albedi

**Affiliations:** 1 Department of Technology and Health, Istituto Superiore di Sanità, Viale Regina Elena, 299, 00161 Roma, Italy; 2 Department of Therapeutic Research and Medicine Evaluation, Istituto Superiore di Sanità, Viale Regina Elena, 299, 00161 Roma, Italy; E-Mail: mariacristina.gaudiano@iss.it; 3 Department of Cell Biology and Neuroscience, Istituto Superiore di Sanità, Viale Regina Elena, 299, 00161 Roma, Italy; E-Mail: fiorella.malchiodi@iss.it

**Keywords:** amyloid oligomers, aggregation, lipid rafts

## Abstract

Amyloid proteins of different aminoacidic composition share the tendency to misfold and aggregate in a similar way, following common aggregation steps. The process includes the formation of dimers, trimers, and low molecular weight prefibrillar oligomers, characterized by the typical morphology of globules less than 10 nm diameter. The globules spontaneously form linear or annular structures and, eventually, mature fibers. The rate of this process depends on characteristics intrinsic to the different proteins and to environmental conditions (*i.e.*, pH, ionic strength, solvent composition, temperature). In the case of neurodegenerative diseases, it is now generally agreed that the pathogenic aggregates are not the mature fibrils, but the intermediate, soluble oligomers. However, the molecular mechanism by which these oligomers trigger neuronal damage is still unclear. In particular, it is not clear if there is a peculiar structure at the basis of the neurotoxic effect and how this structure interacts with neurons. This review will focus on the results we obtained using salmon Calcitonin, an amyloid protein characterized by a very slow aggregation rate, which allowed us to closely monitor the aggregation process. We used it as a tool to investigate the characteristics of amyloid oligomers formation and their interactions with neuronal cells. Our results indicate that small globules of about 6 nm could be the responsible for the neurotoxic effects. Moreover, our data suggest that the rich content in lipid rafts of neuronal cell plasma membrane may render neurons particularly vulnerable to the amyloid protein toxic effect.

## 1. Introduction

The term “amyloid” has been historically referred to misfolded proteins that deposit in tissues as mature fibrils, due to their propensity to aggregate. They are characterized by Congo red- and Thyoflavin T-positivity. In some diseases, such as systemic amyloidosis, these deposits have a pathogenetic role and the disease is caused by the deposition of mature fibrils [1–3]. Until a few years ago, there was a general agreement on the idea that only a limited number of proteins can form “amyloids”. However, it has recently been proposed that “almost every human protein has segments that can form amyloid, the sticky aggregates known for their role in diseases” [4]. As Goldschmidt *et al*. pointed out, all complex proteins have segments with the particular structure that prompts aggregation [5]. These segments are typically about six aminoacid long and they can be exposed when a protein partly unfolds. He speculated that most proteins have evolved so that their structure effectively conceals their amyloid-prone areas. However, this mechanism can be ineffective, due to several factors, with the result of about 50 amyloid-associated diseases.

**Figure 1:**
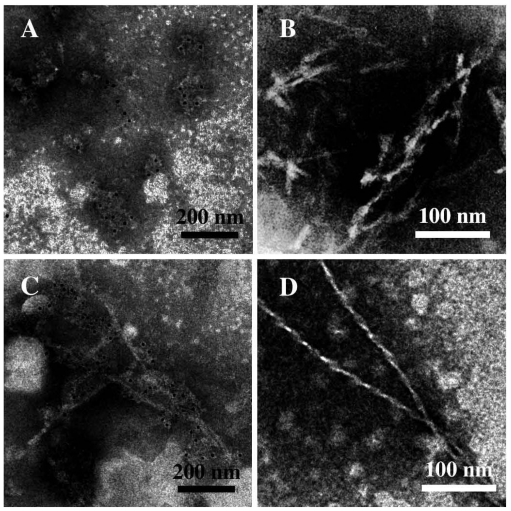
Transmission Electron micrographs showing the characteristic morphology of amyloid aggregates: (**A**) isolated or clustered spherical low molecular weight pre-fibrillar oligomers (PFOs); (**B**) protofibrils; (**C,D**) mature fibers. sCT was identified by immunogold labeling (black dots of 5 nm).

**Figure 2:**
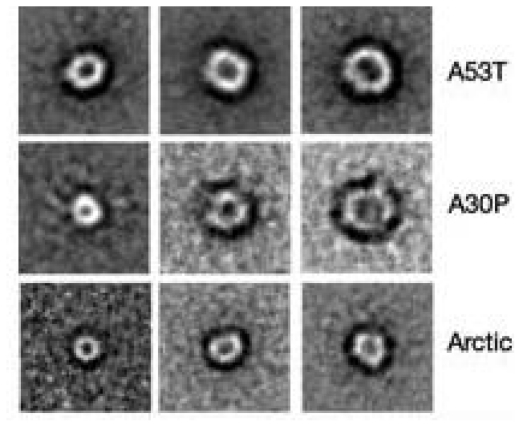
Annular Proto Fibrils (APFs) of amyloid aggregates of mutant α-Synuclein (A53T and A30P) and β-Amyloid (Arctic) proteins imaged by negative staining Transmission Electron Microscopy (courtesy of Lashuel *et al*. [9]).

In the case of neurodegenerative diseases associated with protein misfolding, it is now generally agreed that the pathogenic aggregates are not the mature fibrils, but the intermediate, soluble oligomers [6,7]. Interestingly, oligomers of different amyloid proteins share a surprising structural and functional similarity. First of all, structural investigations, performed by both Transmission Electron Microscopy (TEM) and Atomic Force Microscopy (AFM), showed for all amyloid proteins a common aggregation path influenced by environmental conditions (*i.e.*, concentration, pH, ionic strength, solvent composition, temperature). The process is time-dependent and proceed through several common organization states, including the formation of dimers, trimes, tetramers, low molecular weight Pre-Fibrillar-Oligomers (PFOs) (Figure 1A), linear (Figure 1B,C) or Annular-Proto-Fibrils (APFs) (Figure 2), of about 8–12 nm diameter, to reach the final insoluble fibrillar structure (Figure 1D), in the size micron range, rich in β sheets.

Some authors consider APFs as “off-pathway intermediates” in the process of fibrillization [8] and responsible of the neurotoxicity associated with the formation of amyloid fibrils in Alzheimer’s, Parkinson’s and other age-associated degenerative amyloid diseases [9].

Many studies have been carried out to investigate the relationship between structural features and neurotoxicity of amyloid proteins involved in diseases, such as β-Amyloid (βA) in Alzheimer’s disease (AD), α-Synuclein (αS) in Parkinson’s disease (PD), prion protein in spongiform encephalopaties, inslet amyloid polypeptide in type II diabetes. Noticeably, Bucciantini *et al*. proved that oligomers of synthetic proteins not associated with any disease are also neurotoxic, suggesting that a shared structural feature of the amyloid oligomers is at the basis of neurotoxicity [10]. This result is in good agreement with the general hypothesis that protein misfolding, leading to the exposure of hydrophobic regions of proteins, renders them potentially toxic, independent of their primary sequences. This hypothesis is strongly supported by the observation that a single antibody, specifically designed for βA, is able to recognize soluble oligomers of widely varying primary sequences of amyloid proteins [11].

Misfolded, aggregated proteins have the ability to interact with lipid membranes inducing cell damage and dysfunction, mainly via the formation of ion channels or “pores” [12]. Interestingly, this kind of toxicity, named “hydrophobicity-based toxicity”, is similar to those described for antibacterial toxins and viral proteins [13].

In 2006, Glabe *et al*. proposed that a common core of pathologic pathways exists in the case of amyloid-associated diseases, based on the cellular membrane permeabilization exerted by the amyloid oligomers, independent of the peptide primary sequence [14]. A number of findings support the intriguing hypothesis that amyloid protein toxicity is due to the formation of Ca^2+^-permeable channels in the plasma membrane inducing a dramatic intracellular Ca^2+^ rise, which triggers a cascade of events leading to cell damage and death [11,15].

The pore formation and channel-like activity have been recently described for a number of amyloid proteins, suggesting a possible common mechanism for protein-misfolding diseases. A Ca^2+^-dependent toxicity, based on pore-like protofibrils that cause membrane permeabilization, has also been proposed for αS by Volles *et al*. in PD [16,17].

In 2006, Lanshuel *et al*. [12] suggested that protein fibrillization may be implicated in the pathogenesis of most, if not all, neurodegenerative diseases on the basis of the common structural features of three amyloid proteins: the A30P and A53T αS mutations, associated with PD, and βAARC (arctic) mutation, associated with AD [9]. These proteins were shown to form oligomers rich in β-sheet secondary structure, conformed as annular protofibrils (APFs) with a diameter ranging 7–12 nm substantiating the “amyloid pore” hypothesis. Lin *et al*. showed that βA forms ion channels in lipid bilayers and imaged them for the first time by AFM, suggesting that they were composed of hexamers or tetramers. Moreover, they measured the channel conductivity and tested the channel biological activity on neurons, demonstrating that βA pore formation induces rapid neuritic degeneration and death by a Ca^2+^-dependent toxicity mechanism [18].

Theoretical models have been proposed to predict βA peptide ability to insert in lipid bilayer. Molecular dynamic simulation has been developed for βA ion-channel formation in lipid bilayer on the basis of the amino acid sequence and experimental evidence of multilevel ion-channel conductance. Several pore arrangements can be obtained, all starting from a basic structure composed of a β-hairpin followed by a helix-turn-helix motif [19–22]. Finally, it has been widely demonstrated that the secondary structure of membrane-bound βA and its ability to insert into the lipid membrane are strongly affected by the lipid composition. In particular, the importance of “raft-like” lipid domains in the membrane has been recently pointed out, with particular attention to the presence of gangliosides. In particular, Kakio *et al*. showed that low ganglioside content favors the β-structure conformation [23,24].

The molecular mechanisms by which amyloid oligomers trigger neuronal death are still to be completely understood. In particular, it is not clear what is the peculiar structure at the basis of the neurotoxic effect, how it interacts with the plasma membrane, and if specialized membrane structures, such as the so-called lipid “rafts”, are involved [25–27]. At first, the observation of the particular shape of APFs formed outside the plasma membranes suggested that they could be directly involved in the formation of the “amyloid pore”. This idea was supported by biophysical studies performed using model membranes (liposomes), where several reconstituted amyloid proteins were found to be able to form typical “pores” [11].

However, it has been demonstrated by Kayed *et al*. that preformed APFs are not able to permeabilize lipid membranes, unlike PFOs that bind to membranes. In this paper the authors proposed that APFs formation was due to PFOs assembly driven by interfacial interactions, occurring in the presence of hydrophobic/hydrophilic interfaces, such as lipid bilayers, and that the toxic agents were the PFOs, small globules of about 5 nm diameter, able to form membrane-permeabilizing β-barrel pores [28].

## 2. The Calcitonins

Calcitonin is a polypeptide hormone with 32 amino acids. Salmon calcitonin (sCT) is the form most widely used in therapy [29]; calcitonins from other species have been characterized (including human, sheep, pig, rat, and eel calcitonins) and differ mainly in the sequence of amino acids forming the central portion of the polypeptide. Calcitonin is implicated in calcium homeostasis, reducing bone resorption by inhibiting the release of bone calcium into serum [30]. sCT is administered in the treatment of osteoporosis and other metabolic bone diseases since it arrests bone resorption mediated by osteoclasts. It has been demonstrated that sCT acts via a specific receptor found in many cell types and tissues, suggesting diverse biological roles for CT [31].

Calcitonins are generally considered “amyloid” proteins due to their aggregation behavior, which starts with the formation of oligomers and eventually results in the deposition of fibrils and plaques. Unexpected results were obtained when it was shown that sCT is neurotoxic *in vitro* like other amyloid proteins involved in neurodegenerative pathologies.

Human Calcitonin (hCT) and sCT fibrillation studies based on turbidity measurements showed that both species aggregate over time. Arvinte *et al*. studied the structure of hCT fibrils by TEM, Circular Dichroism (CD) and Fourier Transform Infrared Spectroscopy [32–34]. They proposed a fibrillation model based on a double nucleation mechanism, previously described for gelation of sickle cell hemoglobin [35]. In this model, electrostatic interactions between hCT monomers are responsible for the initial aggregation step (homogeneous nucleation). The resulting aggregates are spherical in shape. This rounded structure could represent the so-called PFOs. In the second step, fibrils grow by fusion of spherical granules (heterogeneous nucleation). The quaternary structure evolution during fibrillation, leading to the largest supramolecular assembly, has been described in detail by Bauer *et al*. in TEM experiments [32]. Arvinte *et al*. demonstrated that hCT molecules have, in the first aggregation step, both α-helical and β-sheet secondary structure components. They proposed that “an intermolecular β-sheet component is formed by the tail residues 23–32”, providing a template for the α-helical roads of residues 8–22 in the fibril formation [33]. In spite of the similarity, hCT tends to aggregate in aqueous solutions more rapidly than sCT, causing the formation of fibrillar precipitates. Differences in the aggregation speed between hCT and sCT were attributed to the different pK values (8.7 and 10.4 for hCT and sCT, respectively) with one positive charge for hCT and three positive charges for sCT at pH 7.4.

In the study on sCT therapeutic activity, its ability to form aggregates has been considered as a reducing factor of its efficacy. In aqueous solution, the peptide is in a random coil conformation, but in the presence of organic solvents (*i.e.*, trifluoroethanol, methanol) or phospholipids, sCT easily acquires an α-helix secondary structure [34,36,37]. sCT has been shown to form amyloid fibrils *in vitro*. sCT aggregation was induced by vapor diffusion in the presence of dimyristoylphosphatidylglycerol (DMPG) at 230 °C [38]. Both Scanning Electron Microscopy and TEM, together with X-ray diffraction, were used. The authors noted the formation of large aggregates and individual long fibers (up to 2000 nm) of 5–6 nm in diameter, very similar to those of βA peptide. X-ray diffraction patterns of the large aggregates strongly suggested that sCT forms cross-β fibrils.

Thus sCT is characterized by a very slow aggregation rate and this property makes it, in our opinion, particularly suitable to be used as a tool to study the molecular mechanisms of amyloid formation and neurotoxicity, with particular attention to the early stages of aggregation, where amyloid oligomers are produced, and to their interaction with cell membranes.

## 3. Our Contribution to the Study of sCT

### 3.1. The Effect of a Mild Oxidation on sCT Aggregation

**Figure 3:**
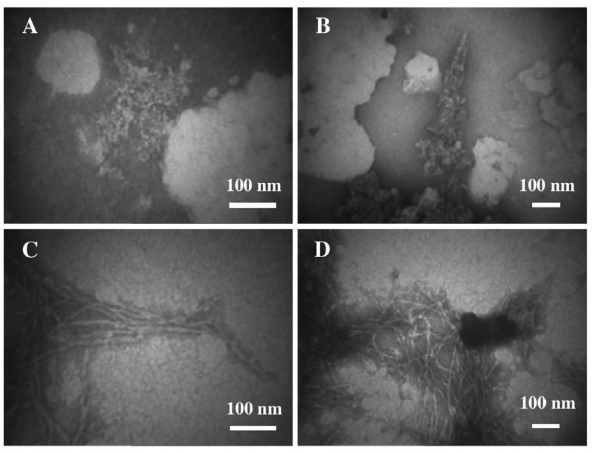
(**A,B**) Transmission Electron Microscopy micrographs (negative staining) relative to the spontaneous aggregation of sCT (after 2 h) at room temperature in phosphate buffer (5 mM). Images show globular aggregates of about 7 nm in diameter (PFOs) organized in disordered clusters (**A**) or in linear structures (**B**); (**C,D**) Micrographs relative to the aggregation, at room temperature (after 2 h) in the presence of free OH radicals following a controlled and gentle oxidization procedure. Images show mature fibers organized in ribbons (**C**) or in large skeins (**D**) (courtesy of Gaudiano *et al*. [39]).

We started investigating sCT in 2003, using reversed phase liquid chromatography (RP-LC), CD and TEM, to characterize the early stages of the aggregation process [39]. TEM images clearly showed globules of about 7 nm in diameter (PFOs), isolated or grouped in clusters but well separated from each other, even if a tendency to form fiber-like aggregates was already observed (Figure 3). The presence of granular and dot-like aggregates was reported, during the first fibrillation step, also in the Arvinte’s paper [33]. As previously reported [34,40], our CD results showed that sCT maintained a random-coil conformation in water solution at pH 7.4 for 2 h.

Moreover, we studied the effects produced by the oxidation process induced by free OH radicals, applying a controlled and mild oxidation procedure (modified Fenton’s reaction [41]), able to simulate the live cell conditions. TEM images relative to sCT in the presence of oxidant mixture clearly showed defined fibers of about 7–8 nm in diameter, organized in large skeins or ribbons (Figure 3).

These images were very similar to those published for hCT by Bauer *et al*. [32] and Arvinte *et al*. [33]. P.J. Gilchrist and J.P. Bradshow observed similar fibrils, for sCT aggregation induced within 9 days by heating the samples at 230 °C in the presence of DMPG [38]. The authors demonstrated a strong similarity between amyloid fibrils of sCT and βA and proposed the cross-β structure for both proteins.

Our RP-LC results [39] showed that OH free radicals induced in sCT the formation of degradation/oxidation products. CD data [39] indicated that the reaction with hydroxyl radicals affected the secondary structure of the peptide, raising the α and β components as well as its aggregation state because of the modifications occurring in the primary sequence, as demonstrated by RP-LC results. The effect observed by CD was more evident in the more concentrated oxidant mixture, indicating that the conformational variations and aggregations depended on hydroxyl radical concentration.

### 3.2. The Effect of a Strong Oxidation and the Presence of Liposomes on sCT Aggregation

In a second paper [42] we focused our investigation on both (i) the *in vitro* time-dependent aging and (ii) chemical oxidation processes of sCT by using hydrogen peroxide as oxidant agent [43,44].

The study was also conducted in the presence of a membrane model consisting of dipalmitoylphosphatidylcholine (DPPC) liposomes, in order to mimic the conformational variations and aggregation processes of sCT occurring in extra-cellular compartment. Liposomes are generally accepted as simplified three-dimensional models of the lipid membranes. They can be easily prepared using plain lipid and structurally characterized in aqueous suspensions by means of spectroscopic techniques or deposited onto suitable substrates for TEM or AFM analysis [45]. The choice of the zwitterionic phosphatidylcholine (PC) was due to its abundance in the actual cellular membranes. Some authors showed that sCT interacts with acidic phospholipids and, in particular, with phosphatidylglycerol (PG), to form water-soluble complexes with sCT in the bilayer [46]. On the contrary, in the presence of dimyristoyl-PC, no strong interactions were observed [37].

It is important to stress that our observations referred to the early stages of the aggregation at very low protein concentration (1.2 × 10^−5^ M) and low ionic strength (Sodium Phosphate Buffer 5 × 10^−3^ M). We observed that the two aggregation rates were very different from each other: very slow (days) in the case of natural time-dependent aging and very fast (minutes) in the presence of hydrogen peroxide.

**Figure 4:**
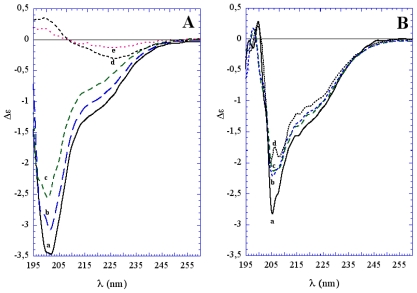
(**A**) Circular Dichroism spectra measured on sCT samples spontaneously aggregating at room temperature in phosphate buffer (5 mM) measured immediately after the sample solution preparation (a) and after 4 (b), 5 (c), 6 (d) and 10 days (e); (**B**) Spectra relative to sCT samples aggregating at room temperature in the presence of hydrogen peroxide immediately after the sample solution preparation (a) and after 4 (b), 5 (c) and 6 days (d) (courtesy of Gaudiano *et al*. [42]).

In the first stage of protein aging in water, the sCT aggregation process occurred with slight conformational changes as demonstrated by CD data (Figure 4A). From the same results, after 6 days an evident conformational change appeared and was accompanied by a more evident aggregation, with a conformation rich in β-sheet structures, as described for mature CT fibrils and plaques.

**Figure 5:**
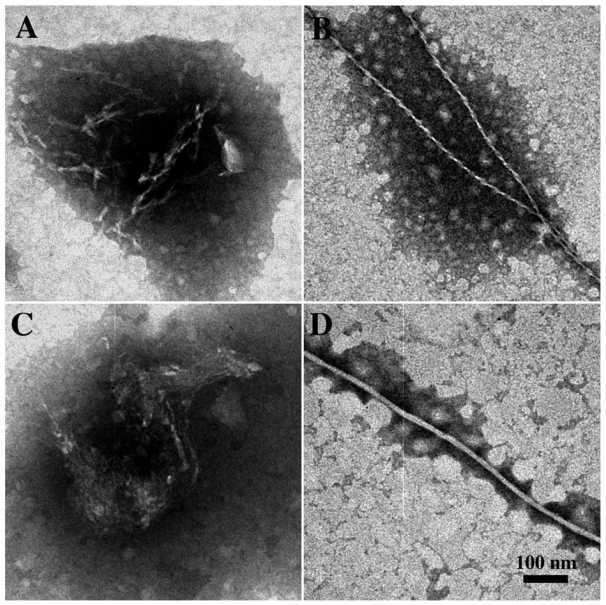
Transmission Electron Microscopy micrographs (negative staining) relative to sCT samples spontaneously aggregating at room temperature in phosphate buffer (5 mM): (**A,B**) immediately after the sample solution preparation and (**C,D**) after a week (courtesy of Gaudiano *et al*. [42]).

TEM images (Figure 5) confirm the sCT aggregation mechanism proposed above. Even at the beginning of the aggregation process, PFOs, protofibrils (A), together with thin cables assembling to form a bigger cable, were observed (B). One week later, fibril ribbons (C) and well-formed big cables (D) were found more frequently.

**Figure 6:**
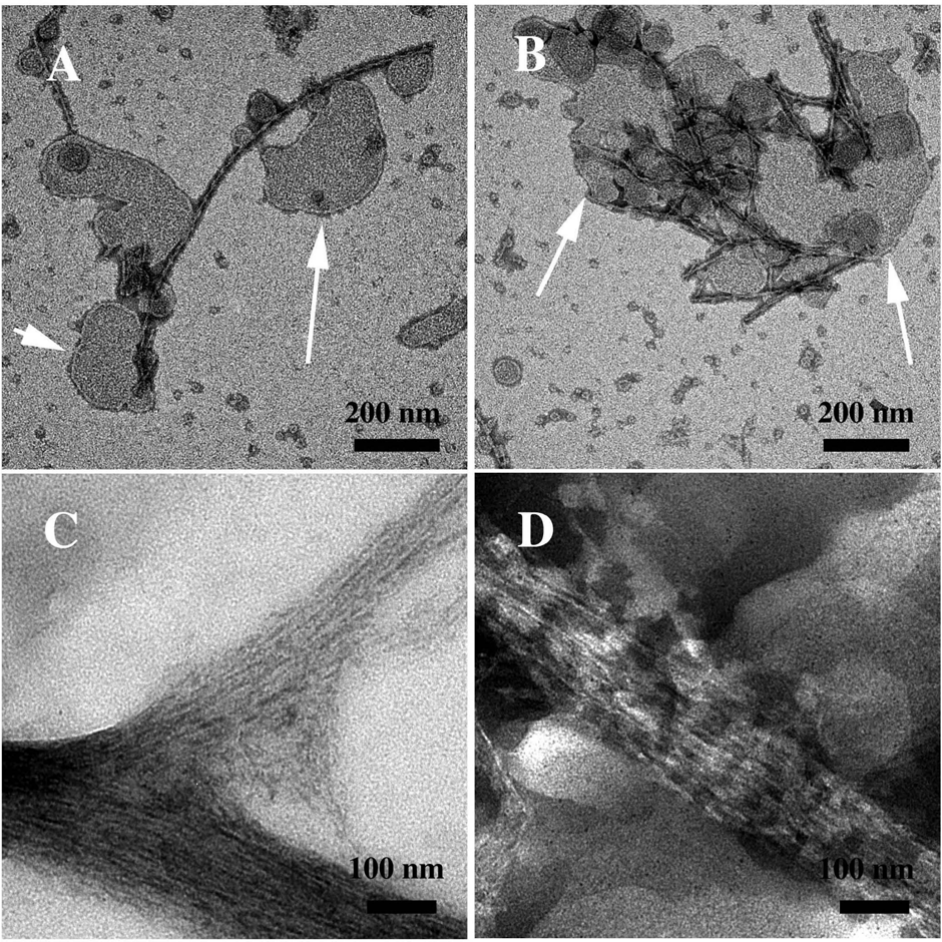
(**A,B**) Transmission Electron Microscopy micrographs (negative staining) relative to the sCT aggregation, in the presence of DPPC liposomes after 6 days, showing an interesting interconnection of liposomes (indicated by arrows) and fibres; (**C,D**) micrographs relative to sCT samples aggregating in the presence of hydrogen peroxide, immediately after the solution preparation, showing large fibril-ribbons (courtesy of Gaudiano *et al*. [42]).

The presence of DPPC liposomes did not seem to considerably affect neither the conformational change at 6 days, nor the aggregation process mainly occurring around liposomes and forming a fibril network (Figure 6).

The chemically-induced oxidation process led to a very fast conformational change different from that occurring during natural aging, as demonstrated by CD data (Figure 4B). TEM images showed that the aggregation was less homogeneous, leading to a “hank” of protein fibrils, both in water solution and in the presence of liposomes (Figure 6).

Chromatographic profiles indicated the formation of a new main product and many other secondary products in the oxidized sample, with the disappearance of the native form [39]. These experiments suggested that the oxidation process affected sCT aggregation and could be an aggregation promoter *in vivo*.

### 3.3. The Effect of Lipid Rafts on sCT Aggregation

**Figure 7:**
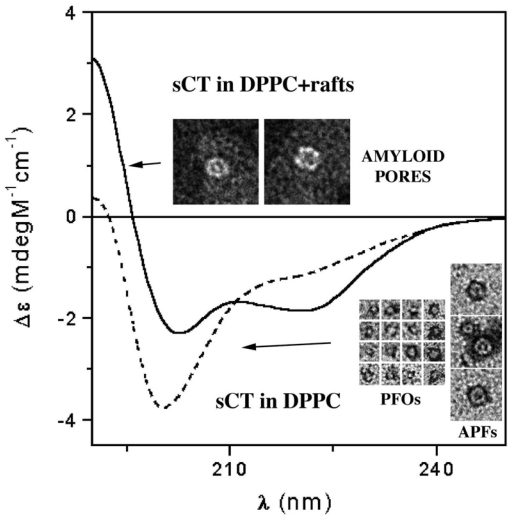
Circular Dichroism spectra of sCT reconstituted in DPPC liposomes containing GM_1_ and Cholesterol, the main components of lipid “rafts” (full line), and in liposomes of plain DPPC (broken line). The presence of lipid “rafts” changes dramatically the protein secondary structure. In the insets Transmission Electron Microscopy images (negative staining) of the sCT aggregates found in the same samples are shown. Amyloid pores were bound to lipid “raft-containing” liposomes, while Pre-Fibrillar-Oligomers (PFOs) and Annular-Proto-Fibrils (APFs) were located outside plain DPPC liposomes.

As stated before, many studies have been carried out to investigate the relationship between structural features and the toxicity of amyloid aggregates. In 2006, we reported for the first time [47], TEM evidence of the spontaneous formation of small sCT globules of less than 10 nm (about 6 nm), namely PFOs. These globules were observed as isolated units or forming complete or incomplete APFs (insets of Figure 7) very similar to those observed for proteins involved in neurodegenerative diseases, such as AD and PD (Figure 2) [9].

We reconstituted sCT in synthetic model membranes of two different kinds: plain DPPC and DPPC with a mixture of GM_1_/cholesterol (GM_1_/chol), which mimicked lipid rafts.

CD experiments performed on sCT in plain DPPC liposomes indicated that the protein, organized in PFOs and APFs, was located outside the liposomes and poorly interacted with lipids, showing limited if any conformational modifications when compared to the sCT monomers (broken line in Figure 7). Conversely, sCT reconstituted in GM_1_/chol-containing liposomes, organized in pore-like structures and localized in the liposomes, dramatically changed its conformation during the interaction with lipids, increasing its β-sheet structure content (full line in Figure 7).

Consistently, immunogold sCT labelling at TEM confirmed that the presence of GM_1_/chol mixture in model membranes strongly improves sCT binding to liposomes [47].

### 3.4. The Effect of sCT Aggregates on Ca^2+^ Flow

Finally, Ca^2+^ influx into liposomes was studied using Fluo-4 dye. We observed that the presence of sCT molecules in plain DPPC liposomes did not affect Ca^2+^ flow through the lipid membranes. Conversely, sCT reconstituted in GM_1_/chol-containing liposomes triggered Ca^2+^ entry through the lipid bilayer.

The all body of our results strongly suggests that sCT forms Ca^2+^-permeable pores only in liposomes containing lipid raft-like mixtures. We observed pore-like structures localized on liposomes at TEM that can be clearly interpreted as amyloid pores (insets of Figure 7). Conversely, in the samples containing plain DPPC liposomes, APFs could never be observed on liposomes. It is worth noting that a dramatic and fast sCT conformational change occurred in this sample while only very small change occurred with plain DPPC liposomes or without liposomes. Thus we can conclude that the pore-like structures observed in the lipid raft-containing liposomes differ from the APFs observed in the other samples (Figure 7) and that the pore formation was triggered by the raft-containing bilayer. It can be speculated that the amyloid pore is formed inside the lipid bilayer, which induces a new sCT conformation rich in β-structures, as expected from several computational studies [19–22]. Our results strongly support the “hydrophobicity-based toxicity” hypothesis and sustain that lipid rafts have a relevant role in the conformational change of sCT oligomers and in their interaction with model membranes.

### 3.5. The Mechanism of Ca^2+^-permeabilization Induced by sCTOs Applied in Cell Cultures

**Figure 8:**
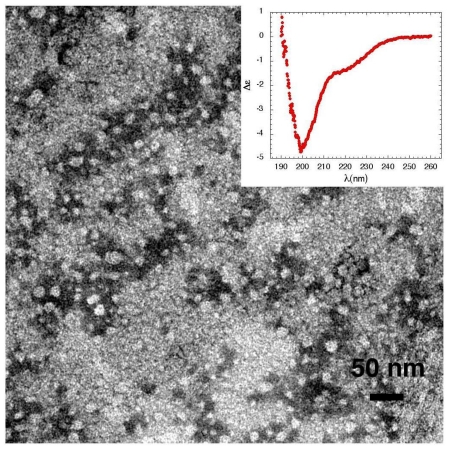
Transmission Electron Microscopy micrograph (negative staining) of sCT Pre-Fibrillar-Oligomers (PFOs) obtained incubating sCT (1 mM) at room temperature, for 3 h. Only globules less than 10 nm in diameter are observed. At this stage of aggregation the Circular Dichroism spectrum (inset) indicates that sCT secondary conformation is still mainly “random”.

To investigate if the mechanism of Ca^2+^-permeabilization induced by sCTOs in synthetic model membranes applies also to living cells and to better clarify the role played by lipid rafts in the sCT pore formation leading to Ca^2+^ unbalance, we extended our work to cell cultures [48], focusing on the peculiar vulnerability of neuronal cells to the toxicity of amyloid proteins. In particular, we chose cell cultures characterized by different lipid rafts content: primary hippocampal neurons at day-*in-vitro* (DIV) 13 (mature neurons), or at DIV 4 (immature neurons), primary rat astrocytic cultures and two immortalized cell lines: MG63 osteoblast-like cells and NIH3T3 fibroblasts. sCT was dissolved in phosphate buffer and incubated for 3 h at room temperature to allow the formation of sCTOs. Samples were characterized at both TEM and CD. sCT formed small isolated globules of mean diameter of about 10 nm (Figure 8). No mature fibrils were observed and CD data (inset) showed that the sCT conformation was mainly random coil in good agreement with results reported above.

**Figure 9:**
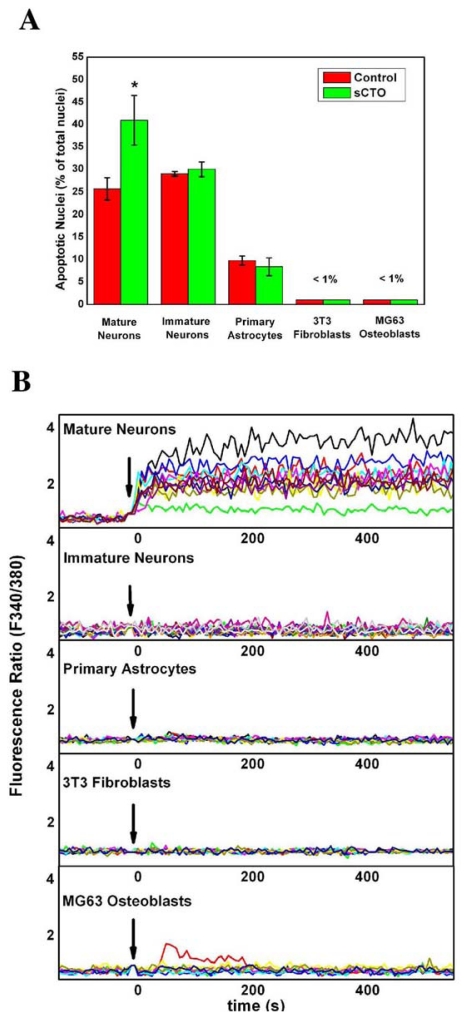
(**A**) Apoptosis percentages in different cell types after treatment with sCT oligomers. The effect is evident only in mature hippocampal neurons. Apoptosis was evaluated by staining nuclei with Hoechst 33258; (**B**) At the same time, sCTO induces increase in intracellular Ca^2+^ levels in mature hippocampal neurons, but not in immature neurons, primary astrocytes, 3T3 fibroblasts and MG3 osteoblasts. Ca^2+^ levels were evaluated by optical fluorimetric recordings with Fura-2AM. Each color is associated to a single cell of the sample.

**Figure 10:**
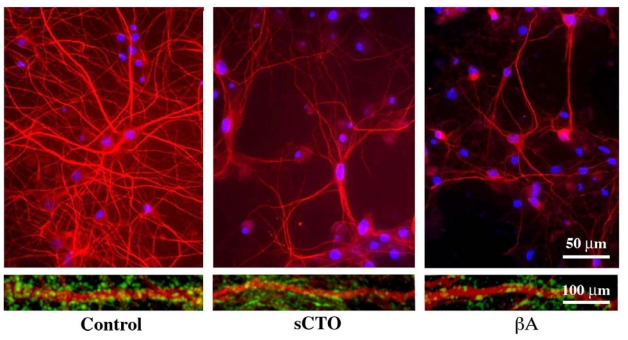
sCT and βA oligomers similarly damage the neuritic tree and synapses in mature hippocampal neurons. After treatments, the extension of the dendritic tree, immunolabelled for MAP2 (red fluorescence) is evidently reduced, while the number of synapses, immunolabelled for synaptophysin (green fluorescence) is decreased (courtesy of Malchiodi-Albedi *et al*. [48]).

After treatment with sCTOs, we measured the Ca^2+^ influx by the FURA-2AM method in all cell types and determined the toxic effect in neuronal cultures by analysing apoptotic rate by the Hoechst 33258 stain and the TUNEL assay [48]. Treated with sCTOs, mature hippocampal neurons showed a marked increase in the apoptotic rate with respect to controls (Figure 9A). The neuritic tree and synapses was also severely damaged. As a whole, these effects are reminiscent of those induced by βA (Figure 10). No modification of apoptotic rate was observed in the other cell types (immature hippocampal neurons, primary astrocytes, 3T3 fibroblasts and MG3 osteoblasts). Results on Ca^2+^ influx (Figure 9B) clearly showed that mature hippocampal neurons had an intense and sustained increase in cytosolic Ca^2+^ induced by sCTOs, which involved nearly the entire population of the recorded cells.

Immature hippocampal neurons, as well as primary astrocytes, 3T3 fibroblasts and MG3 osteoblasts, failed to show such a dramatic increase in the cytosolic Ca^2+^ content after the same treatment with sCTOs. Depletion of intracellular Ca^2+^ stores with thapsigargin did not affect sCTO-induced Ca^2+^ rise, suggesting that it was mostly due to an extracellular Ca^2+^ influx. MK801, a specific NMDA inhibitor, as well as antibodies against the NR1subunit of NMDA receptor, failed to affect sCTO-driven Ca^2+^ influx, suggesting that the NMDA receptor was not involved. On the contrary, pre-treatment with an antibody against the ganglioside GM_1_, aimed at blocking LRs, completely abolished sCTOs-induced Ca^2+^ increase. These results led us to hypothesize that the different response to sCTOs in mature hippocampal neurons could be due to a different content in lipid rafts. Measure of the weight ratio between cholesterol in LRs (LDTI) and total cholesterol confirmed that plasma membrane of mature hippocampal neurons had a much higher content in LRs than the other cell types. It has also been demonstrated that LRs increase in the plasma membrane during *in vitro* maturation in hippocampal neurons [49]. This could explain why immature neurons were insensitive to sCTO toxicity. Thus, content in LRs higher than the other cell types could render neurons more vulnerable to amyloid toxicity. To further corroborate this hypothesis, we manipulated LRs in mature neuronal cells in the attempt of modifying sCTO-induced intracellular Ca^2+^ entry. Pre-treatment of neurons with an antibody against GM_1_, a ganglioside particularly abundant in LRs, completely suppressed sCTO-driven Ca^2+^ rise, without altering NMDA receptor activity. Furthermore, LR disruption obtained by neuraminidase, which removes sialic acid from gangliosides, inhibited Ca^2+^ rise and protected against sCTO neurotoxicity, probably modifying the plasma membrane area, rich in lipid raft, that is susceptible to the insertion of the pore-like structures.

These results strongly support the conclusion that the intense and protracted Ca^2+^ dysregulation observed after sCTOs treatment is reliably due to the pore formation in a particularly suitable environment, *i.e.*, the LR-rich neuronal plasma membrane.

## 4. Concluding Remarks

The use of sCT as a tool for the investigation of amyloid aggregation allowed us to follow the oligomer formation from its very early stages, in an oxidized or not environment. In the presence of oxidizing agents, the aggregation is very fast, and no oligomers of small molecular weight can be observed, while long mature fibres predominate. Consistently, the sCT secondary structure immediately changes in a β-rich conformation. After this structural change, the process remains stationary.

Conversely, natural, time-dependent aging leads to the formation of typical amyloid oligomers of small molecular weight, which spontaneously organize in linear or annular clusters made of several units. However, the morphological modifications observed at this stage are not accompanied by any important conformational change, the secondary structure persisting mainly as random coil, as in the monomer. Only after a long period does natural aging lead to the formation of protofibrils and eventually to mature fibres, characterized by the well-known β-rich conformation. The sCT behavior is therefore very similar to that of other important amyloid proteins (βA, αS and Pr), supporting the hypothesis that a common mechanism exists for amyloid oligomer formation.

The interaction of sCT molecules with model membranes clearly demonstrates that only in the presence of lipid rafts is the protein incorporated in the lipid bilayer and that this incorporation leads to dramatic conformational change towards β-rich structure. More important, this change was correlated to the observation of membrane permeabilization and pore-like structures.

These results sustain the idea that the sCT structures formed into the lipid bilayer were very different from the APFs formed outside the membranes. This idea is supported by results from Kayed laboratory [28], which showed that βA APFs did not permeabilize cell membranes, while PFOs did.

Thus, in our opinion, PFOs, *i.e.*, the small globules of about 6 nm formed by practically all amyloid proteins, could be responsible for the amyloid-pore formation. They could be incorporated in the bilayer due to electrostatic attraction with charged lipid rafts components, such as gangliosides, and aggregate inside the lipid bilayer forming the amyloid pore characterized by β-rich structure.

The results of the experiments performed on different cell types confirmed this hypothesis, since the high content in lipid rafts of the plasma membrane of mature hippocampal neurons was responsible of Ca^2+^ influx after sCTO treatment. Our results point to the importance of lipid composition of cell membrane in the mechanisms of toxicity of amyloid proteins.
